# High expression of phosphoglycerate dehydrogenase predicts poor outcome in patients with high-grade serous ovarian cancer

**DOI:** 10.1093/oncolo/oyae161

**Published:** 2024-06-26

**Authors:** Lilian van Wagensveld, Tom Van Nyen, Daniela Annibali, Gabe S Sonke, Roy F P M Kruitwagen, Frederic Amant, Hugo M Horlings

**Affiliations:** Department of Research and Development, Netherlands Comprehensive Cancer Organization (IKNL), 3511 DT Utrecht, The Netherlands; Department of Molecular Pathology, The Netherlands Cancer Institute, 1066 CX Amsterdam, The Netherlands; GROW, School for Oncology and Reproduction, 6229 HX Maastricht, The Netherlands; Gynecological Oncology Laboratory, Department of Oncology, KU Leuven and Leuven Cancer Institute (LKI), 3000 Leuven, Belgium; Division of Oncogenomics, The Netherlands Cancer Institute, 1066 CX Amsterdam, The Netherlands; Gynecological Oncology Laboratory, Department of Oncology, KU Leuven and Leuven Cancer Institute (LKI), 3000 Leuven, Belgium; Department of Gynaecologic Oncology, The Netherlands Cancer Institute, 1066 CX Amsterdam, The Netherlands; Department of Medical Oncology, The Netherlands Cancer Institute, 1066 CX Amsterdam, The Netherlands; GROW, School for Oncology and Reproduction, 6229 HX Maastricht, The Netherlands; Department of Obstetrics and Gynecology, Maastricht University Medical Centre, 6229 HX Maastricht, The Netherlands; Gynecological Oncology Laboratory, Department of Oncology, KU Leuven and Leuven Cancer Institute (LKI), 3000 Leuven, Belgium; Department of Gynaecologic Oncology, The Netherlands Cancer Institute, 1066 CX Amsterdam, The Netherlands; Department of Obstetrics and Gynecology, University Hospitals Leuven and Department of Oncology, 3000 Leuven, Belgium; Department of Molecular Pathology, The Netherlands Cancer Institute, 1066 CX Amsterdam, The Netherlands

**Keywords:** phosphoglycerate dehydrogenase/metabolism, ovarian neoplasms/pathology, humans, carcinoma, ovarian epithelial/drug therapy, female

## Abstract

**Introduction:**

High-grade serous ovarian cancer (HGSOC) is characterized by high mortality and prevalent recurrences. This study investigates the prognostic value of phosphoglycerate dehydrogenase (PHGDH) in HGSOC which has been linked to metabolic reprogramming and recurrences in other cancers.

**Methods:**

Data from 306 patients with advanced-stage HGSOC treated between 2008 and 2015 were analyzed. PHGDH expression levels were determined using immunohistochemistry and categorized as “low” or “high.”

**Results:**

PHGDH-high was associated with higher FIGO stage and increased use of neoadjuvant chemotherapy. Patients with PHGDH-high tumors had significantly worse survival than PHDH-low, even after adjusting for confounding factors.

## Teaser

These findings highlight the potential of phosphoglycerate dehydrogenase as a prognostic biomarker in high-grade serous ovarian cancer and suggest its involvement in metabolic reprogramming and progression. Targeting the PHGDH pathway could be used as a therapeutic strategy in primary tumors and recurrences. Further validation is necessary to explore its clinical utility as new treatment modalities evolve, possibly offering personalized treatment.

## Introduction

**Figure 1.**
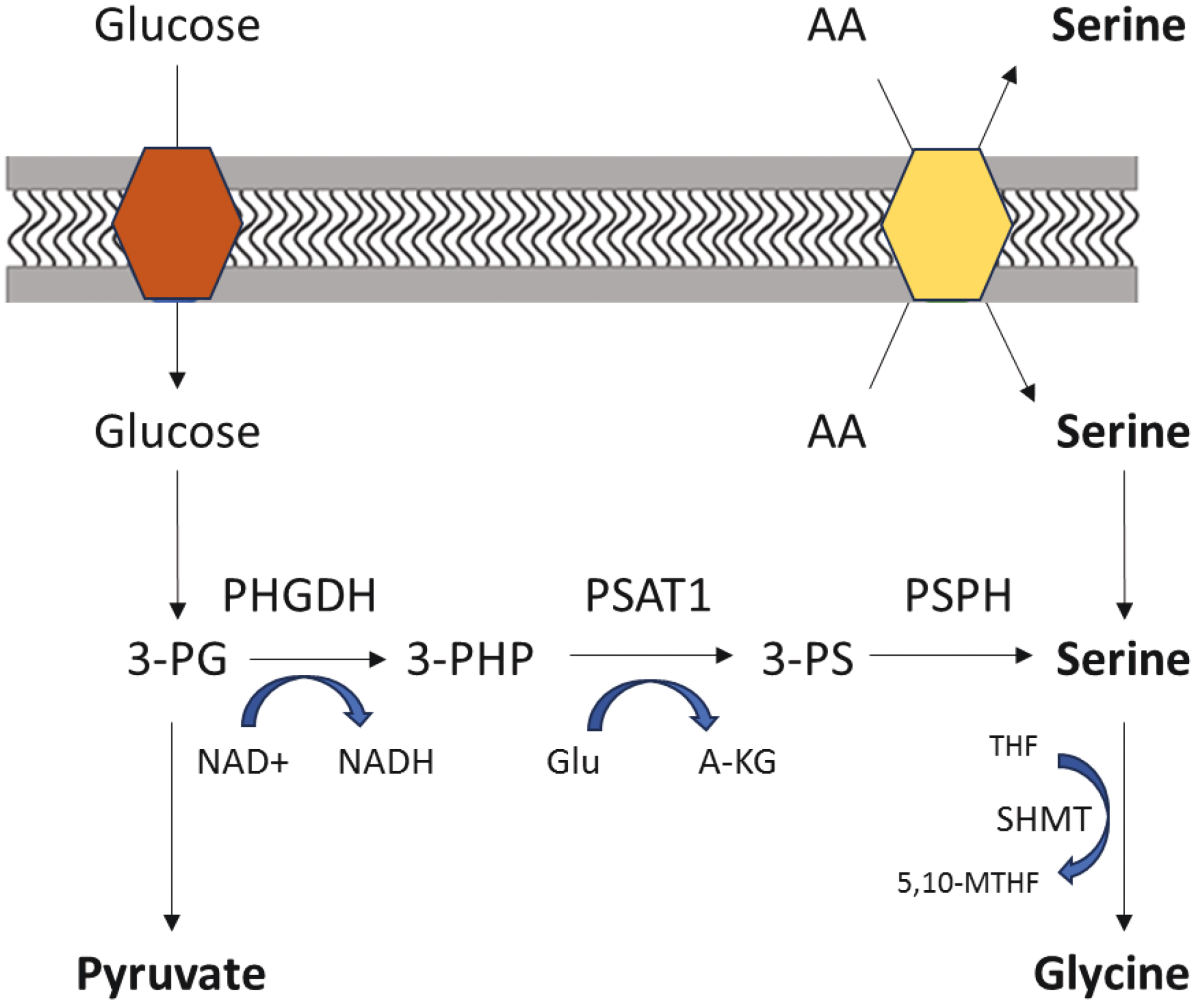
Serine synthesis pathway (SSP). Serine can either be imported from the extracellular space via amino acid (AA) exchangers or synthesized de novo from glucose. The enzyme PHGDH, acting as the rate-limiting step, catalyzes the oxidation of 3-phosphoglycerate (3-PG) to 3-phosphohydroxypyruvate (3-PHP) using NAD+ as a cofactor. Subsequently, phosphoserine aminotransferase (PSAT1) transaminates 3-PHP to 3-phosphoserine (3-PS) and α-ketoglutarate (α-KG) into the tricarboxylic acid cycle using glutamate (Glu) as nitrogen donor. Lastly, phosphoserine phosphatase (PSPH) hydrolyzes 3-PS to serine. Serine is further metabolized by hydroxymethyltransferase (SHMT) into glycine and 5,10-methylenetetrahydrofolate (5,10-MTHF), with tetrahydrofolate (THF) supplying methyl groups.

Ovarian cancer is the deadliest gynecological malignancy in Western countries,^1^ with high-grade serous (HGSOC) being the most prevalent subtype. Standard treatment is platinum-based chemotherapy combined with debulking surgery^2^; however, the vast majority of patients develop recurrent disease. Metabolic reprogramming is linked to malignant proliferation and recurrent disease^3^ and is emerging as a possible source of therapeutic targets. The de novo serine synthesis pathway (SSP; Figure 1) plays a crucial role in tumor metabolic adaptation and carcinogenesis and is mediated by phosphoglycerate dehydrogenase (PHGDH).^4^ The prognostic value of PHGDH has been shown in multiple cancers,^5^ yet little is known about its role in HGSOC. In this study, we examined PHGDH as a prognostic biomarker of survival in HGSOC.

## Methods

**Figure 2.**
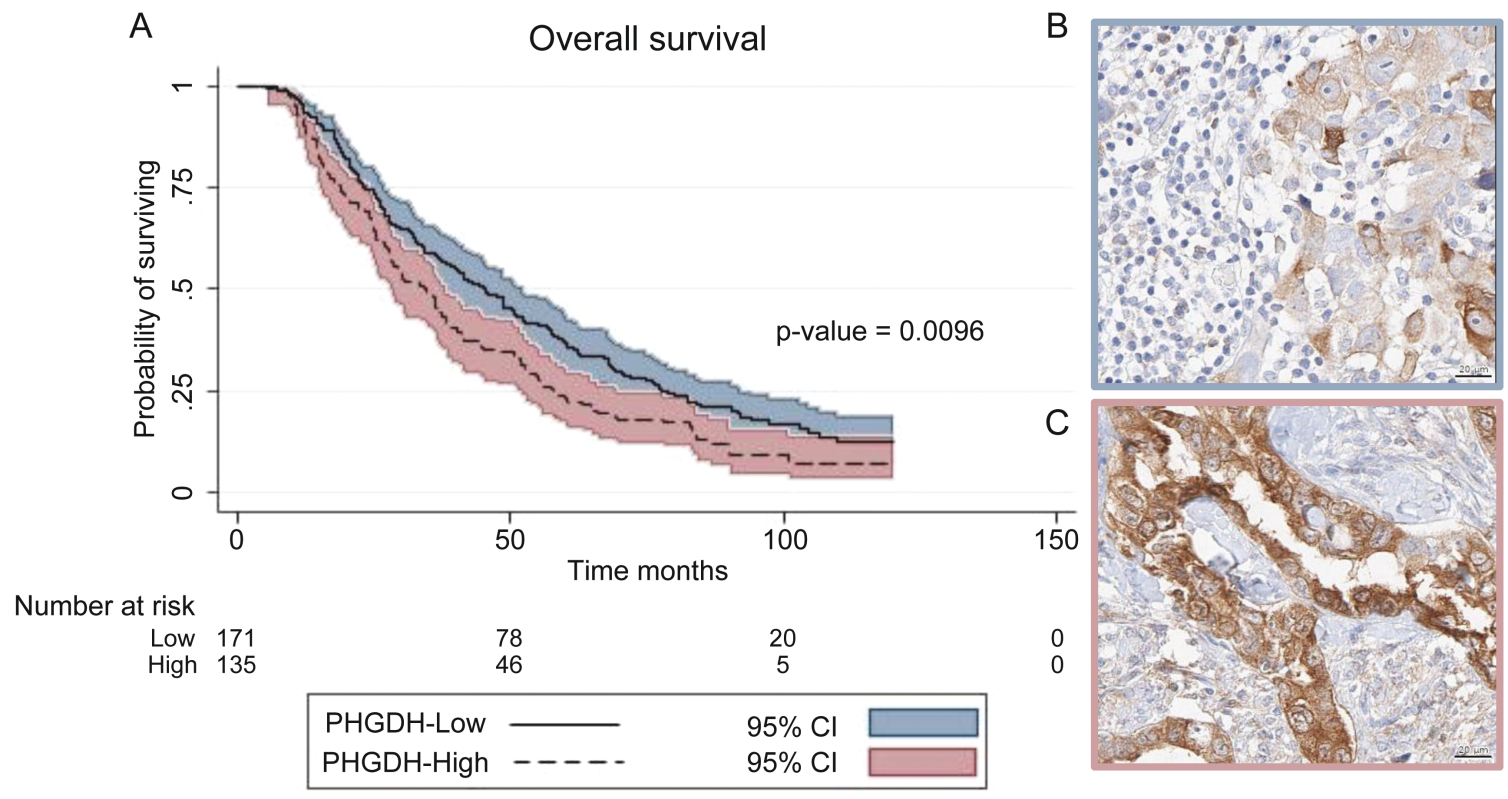
Survival analyses of patients with HGSOC, according to PHGDH levels. (A) Kaplan-Meier curves for overall survival according to PHGDH-high and PHGDH-low. *P* values were derived with the use of the log-rank statistic. (B) Immunohistochemical (IHC) staining of a patient with low PHGDH tumors. (C) IHC staining of a patient with high PHGDH tumors.

We included 322 women with FIGO stage 2b-4 HGSOC who received either primary debulking surgery followed by adjuvant chemotherapy or neoadjuvant chemotherapy and interval debulking (NACT-IDS), in one of 3 Dutch referral hospitals (Netherlands Cancer Institute (NKI-AVL), Maastricht University Medical Center and Amsterdam University Medical Center) between 2008 and 2015. Patient data were extracted from the Netherlands Cancer Registry (NCR). Pathological data and tumor blocks were obtained from the Dutch Nationwide Pathology Databank (PALGA). Institutional review board approval of the NCR (K19.074), PALGA (2019-169), and NKI-AVL (CFMPB297) was obtained. The dataset was anonymous and exempt from ethics review board approval. All cases underwent pathological review by 3 dedicated pathologists (K.V.d.V., H.H, and J.S.). PHGDH was detected by immunohistochemistry (HPA021241, Sigma Aldrich) and scored by multiplying staining intensity (0, no; 1, weak; 2, moderate; and 3, intense staining) with the percentage of positive cells (0%-100%). PHGDH was subgrouped into “low” and “high” based on the cutoff point with the most discriminative survival power (PHGDH≥210; Figure 2B, 2C). Pathologists were blinded to patient characteristics. Characteristics are summarized using descriptive statistics and compared at PHGDH level using the chi-square test, *t*-tests, and Wilcoxon rank-sum tests. Kaplan-Meier survival estimates, log-rank tests, and Cox regressions were used to assess survival differences between low and high PHGDH. Analyses were conducted using STATA/SE (version 14.1, STATA-CORP). Two-sided *P* <.05 was considered statistically significant.

## Results

Tumors from 306 patients were available for analysis. Median PHGDH level was 200 (interquartile range [IQR] = 160-270) resulting in 133 PHGDH-high tumors. PHGDH-high tumors were associated with a higher FIGO stage compared to PHGDH-low tumors, yet not significantly (stage 4; 33% vs 24%, *P* = .13). NACT was more often administered to patients with PHGDH-high tumors (63.7% vs 48%, *P* = .006; Table 1). The remaining characteristics were similar between high and low PHGDH. PHGDH-high was associated with an unfavorable survival compared to PHGDH-low (median survival: 35 months vs 45; hazard ratio (HR) = 1.38; 95% CI, 1.01-1.76; *P* = .01; Figure 2A), which remained similar after adjustment for FIGO stage, treatment sequence and debulking status (HR = 1.29; 95% CI, 1.00-1.66; *P* = .05).

**Table 1.** Patient and tumor characteristics.

Characteristics	Low (*n* = 171)	High (*n* = 133)	*P*		Low (*n* = 171)	High (*n* = 133)	*P*
Therapy				Age (years)			
PDS	89 (52.0%)	49 (36.3%)	.006	<65	69 (40.4%)	62 (45.9%)	.27
NACT	82 (48.0%)	86 (63.7%)		65-75	75 (43.9%)	47 (34.8%)	
				>75	27 (15.8%)	26 (19.3%)	
Residual disease				FIGO stage			
Suboptimal	14 (8.2%)	12 (8.9%)	.66	FIGO 2	12 (7.0%)	5 (3.7%)	.13
Optimal	52 (30.4%)	48 (35.6%)		FIGO 3	114 (66.7%)	83 (61.5%)	
Complete	99 (57.9%)	73 (54.1%)		FIGO 4	41 (24.0%)	45 (33.3%)	
Unknown	6 (3.5%)	2 (1.5%)		Unknown	4 (2.3%)	2 (1.5%)	
Chemotherapy regimen				Metastases, location^*^			
Carboplatin and paclitaxel	149 (87.1%)	112 (83.0%)	.38	Pleural effusion	19 (11.1%)	21 (15.6%)	.98
Carboplatin,paclitaxel and ≥1 other chemotherapeutics	3 (1.8%)	3 (2.2%)		Lymphnodes	7 (4.1%)	8 (5.9%)	
Carboplatin based	7 (4.1%)	12 (8.9%)		Visceral	8 (4.7%)	7 (5.2%)	
Platinum free	7 (4.1%)	5 (3.7%)		Other	6 (3.5%)	7 (5.2%)	
Unknown	5 (2.9%)	3 (2.2%)					
Number of Cycles				Charlson comorbidity			
<6 cycles	15 (8.8%)	11 (8.1%)	.62	Charlson 0	109 (63.7%)	97 (71.9%)	.22
6 cycles	131 (76.6%)	98 (72.6%)		Charlson 1-2	55 (32.2%)	36 (26.7%)	
>6 cycles	17 (9.9%)	18 (13.3%)		Charlson≥3	2 (1.2%)	0 (0.0%)	
Unknown	8 (4.7%)	8 (5.9%)		Unknown	5 (2.9%)	2 (1.5%)	
Ascites				CA-125			
≥100mL	42 (24.6%)	39 (28.9%)	.92	>35	127 (74.3%)	104 (77.0%)	.81
Unkown	35 (20.5%)	11 (8.1%)		Unknown	41 (24.0%)	28 (20.7%)	
Mandart score				BRCA mutation			
1-2	15 (8.8%)	16 (11.9%)	.100	No mutation	138 (80.7%)	98 (72.6%)	.35
3	26 (15.2%)	40 (29.6%)		BRCA1 mutation	18 (10.5%)	20 (14.8%)	
4-5	41 (24.0%)	30 (22.2%)		BRCA2 mutation	13 (7.6%)	13 (9.6%)	
Not applicable	89 (52.0%)	49 (36.3%)		Unknown	2 (1.2%)	4 (3.0%)	

PHGDH level (median = 200, IQR [160-270]; median intensity = 3, IQR [2-3]; median percentage = 90, IQR [70-100]).

^*^Only in patients with metastasis.

Abbreviations: PDS; primary debulking surgery, NACT; neoadjuvant chemotherapy, IQR; interquartile range.

## Discussion

The biosynthesis of serine, mediated by PHGDH, has been linked to the development and malignant proliferation of multiple cancer types.^5^ Serine is either acquired through uptake or synthesized via the SSP (Figure 1). The glycine, one-carbon, serine network produces carbon units essential for various metabolic pathways, such as the anabolic metabolism, maintaining redox balance, and shaping the epigenetic landscape.^6^ Our data portray that high PHGDH levels in primary tumors at initial diagnosis are associated with worse survival in HGSOC. Interestingly, in a prior study, in which we investigated PHGDH levels in vivo and in vitro, we depicted that in a subgroup of HGSOC a downregulation of PHGDH occurred when the disease recurred after primary treatment and became platinum resistant.^7^ Taken together, a dynamic role of PHGDH could be hypothesized in which higher levels at diagnosis are associated with unfavorable prognosis and metabolic adaptation could result in lower PHGDH levels in platinum-resistant recurrences. The PHGDH pathway could reveal possible therapeutic targets in primary tumors, per instance by targeting enzymes involved in the PHGDH pathway in tumors with elevated PHGDH expression, as well as vulnerabilities in resistant recurrences. Nonetheless despite the completeness in patient data and large size, the current study is not without limitations. Such limitations are its historical nature, heterogenic patient group and lack of validation cohort. Therefore, PHGDH’s role in HGSOC and possible influence on survival should be further validated. As new treatment options emerge, understanding underlying mechanisms of cancer progression and chemoresistance may assist in patient tailored treatment.

## Data Availability

Data sharing of anonymous clinical data from the NCR will be considered for noncommercial, research, or statistical-based use on a case-by-case basis (to be requested and approved by the NCR; gegevensaanvraag@iknl.nl). The human sequence data and tumor tissue data generated in this study are not publicly available due to patient privacy requirements but are available upon reasonable request from the corresponding author.
